# Targeting the lung innate pathways during tuberculosis can improve vaccine-induced protection via Th17 responses in diversity outbred mice

**DOI:** 10.1128/mbio.03232-25

**Published:** 2026-01-20

**Authors:** Mushtaq Ahmed, Shibali Das, Bruce A. Rosa, Javier Rangel Moreno, Deepak Kaushal, Makedonka Mitreva, Shabaana A. Khader

**Affiliations:** 1 Department of Microbiology, The University of Chicago 2462, Chicago, Illinois, USA; 2 Department of Molecular Microbiology, Washington University in St. Louis 7548, St. Louis, Missouri, USA; 3 Department of Medicine, Washington University in St. Louis 7548, St. Louis, Missouri, USA; 4 Department of Medicine, Division of Allergy, Immunology and Rheumatology, University of Rochester Medical Center 6927, Rochester, New York, USA; 5 Southwest National Primate Research Center, Texas Biomedical Research Institute, San Antonio, Texas, USA; Universiteit Gent, Gent, Belgium

**Keywords:** *Mycobacterium tuberculosis*, vaccines, Th17 cells

## Abstract

Tuberculosis (TB), caused by the bacterium *Mycobacterium tuberculosis* (*Mtb*), infects approximately one-fourth of the world’s population. Inbred mouse models of TB do not reflect the pathological states and heterogeneity seen in human TB disease. Thus, we recently established a model of TB in diversity outbred (DO) mice, which displayed heterogeneity in inflammatory and protective responses following aerosol *Mtb* infection. In the current study, we show that DO mice vaccinated with *M. bovis* Bacille Calmette Guerin (BCG) are significantly protected upon *Mtb* HN878 infection, and protection is associated with the induction of transcriptional pathways involved in transforming growth factor B (TGF-β) and Toll-like receptor (TLR)-10 signaling. Targeting lung innate pathways in BCG-vaccinated DO mice using adjuvants also further improved protection upon *Mtb* infection by inducing genes associated with cellular responses to external stimuli, B-cell responses, as well as IL-17-producing CD4^+^ T-cell responses. Depletion of CD4^+^ T cells resulted in loss of vaccine-induced protection in DO BCG-vaccinated and adjuvant-treated *Mtb*-infected mice. Together, our new results show that innate targeting of the lung by activating TLR pathways could induce protective pathways in T cells that significantly improve upon the protection induced by BCG vaccination. Additionally, the DO mouse model of vaccination and *Mtb* infection can provide novel insights into immune pathways that are important for improving vaccine-induced protection against TB.

**IMPORTANCE:**

Bacille Calmette Guerin (BCG) vaccination in genetically diverse outbred (DO) mice provides significant protection against *Mycobacterium tuberculosis* (*Mtb)* challenge. This protection induced pathways associated with transforming growth factor B (TGF-β) receptor complex, genes associated with lung repair, and Toll-like receptor (TLR)-10 pathway. The enhanced protection observed in BCG-vaccinated mice correlated with improved formation of B-cell follicles and IL-17-producing CD4^+^ T-cell responses. CD4^+^ T-cell responses mediated the enhanced protection in the lungs of DO mice vaccinated with BCG + adjuvant, as depletion of CD4^+^ T-cell responses reversed the enhanced protection. The DO mouse model of tuberculosis vaccination is a highly relevant model to probe mechanisms of vaccine-induced protection and provide novel insights into lung pathways that mediate protection. The study also found that genes associated with lung repair, including TGF-β receptor complex pathways, were induced in BCG-vaccinated *Mtb*-infected DO mouse lungs. The study suggests that the activation of lung innate pathways in BCG vaccination through the use of mucosal Amph CpG delivery, CD40L activation, and IL-10 neutralization could significantly enhance protection upon *Mtb* challenge.

## INTRODUCTION

Tuberculosis (TB), caused by the bacterium *Mycobacterium tuberculosis* (*Mtb*), infects approximately one-fourth of the world’s population and with 1.6 million deaths globally each year (1). While a majority of infected persons remain asymptomatic (controllers), *Mtb* infection can progress to active clinical disease in TB progressors. The immune mechanisms that mediate progression from infection to TB disease, or *Mtb* control, are not clearly defined. A more comprehensive understanding of immune factors correlating with risk of disease progression, as well as protection against TB, is necessary for targeting new pathways to promote immune control of *Mtb*. Animal models, especially inbred mice, are a significant model that has been used to delineate mechanisms of immunity and inflammation in TB. However, inbred mouse models of TB do not reflect the pathological states and heterogeneity seen in human TB disease. Thus, we have recently established a model of TB in diversity outbred (DO) mice, which displayed broad heterogeneity in inflammatory and protective responses following aerosol *Mtb* infection (2). DO mouse populations infected with *Mtb* HN878, a hypervirulent clinical *Mtb* strain, either display contained infection (controllers) or progress to chronic inflammatory TB disease (progressors) (2). We have shown that disease-induced gene expression signatures in the lungs of progressor DO mice translate across species and overlap with progressor transcriptional signatures in human blood and non-human primate lungs (2). Studies from other groups show that the DO mice infected with the *Mtb* Erdman strain also exhibit disease heterogeneity and provide a spectrum of *Mtb* infection (3). Cumulatively, the DO mouse model in TB has also helped identify biomarkers such as *S100A8/A9* and *CXCL1* (4, 5), which are associated with the progression of TB and serve as a useful platform to study host parameters of *Mtb* infection. A recent study has shown that DO mice vaccinated with *M. bovis* Bacille Calmette Guerin (BCG) provided protection upon *Mtb* challenge, maintained heterogeneity in disease outcomes, and survived longer than DO mice than unvaccinated but *Mtb*-infected DO mice (6). Additionally, mice that received intravenous BCG were better able to control *Mtb* infection when compared with intradermal BCG-vaccinated DO mice (7). Thus, DO mice could also serve as improved small-animal models for evaluation of vaccine candidates and for delineating TB vaccine-induced mechanisms of protection.

In the current study, utilizing the DO mouse model of vaccination and TB, we first determined immune features associated with BCG vaccine-induced protection against TB. Our studies demonstrate that BCG-vaccinated DO mice are significantly protected against *Mtb* HN878 infection and exhibit substantial heterogeneity in protective outcomes. This protection is associated with the induction of transcriptional pathways involved in innate immune and cytokine signaling that are shared with *Mtb*-infected mice, while also uniquely engaging pathways related to lung repair, transforming growth factor–β (TGF-β) signaling, and the Toll-like receptor (TLR)-10 cascade. Our prior studies had shown that targeting innate immune pathways in the lung by activating TLR and CD40 activation pathways at the time of *Mtb* infection could further improve *Mtb* control in BCG-vaccinated inbred C57BL/6 mice (8). Therefore, to assess whether we can improve upon BCG-induced protection in a genetically heterogeneous DO mouse population, individual DO mice were vaccinated with BCG and rested, at the time of infection received mucosal delivery of adjuvant (monophosphoryl lipid A [MPL]) a TLR4 agonist, CD40 agonist, along with IL-10 neutralization and immunodominant *Mtb* antigen, Antigen 85B (Ag85B). This adjuvant treatment in BCG-vaccinated DO mice significantly improved protection upon *Mtb* infection with induction of pathways associated with cellular responses to external stimuli, B-cell responses as well as IL-17-producing CD4^+^ T-cell responses. Indeed, depletion of CD4^+^ T cells resulted in loss of vaccine-induced protection in DO BCG-vaccinated *Mtb*-infected mice. Together, our new results described here show that innate targeting of the lung by activating TLR pathways could induce protective T-cell-mediated pathways that improve upon the protection induced by BCG, even in genetically diverse hosts. Additionally, the DO mouse model of vaccination and *Mtb* infection can provide novel insights into innate and adaptive immune pathways that are important for improving vaccine-induced protection against TB.

## RESULTS

### Innate targeting of the lung improves BCG-induced protection in DO *Mtb*-infected mice

**Fig 1:**
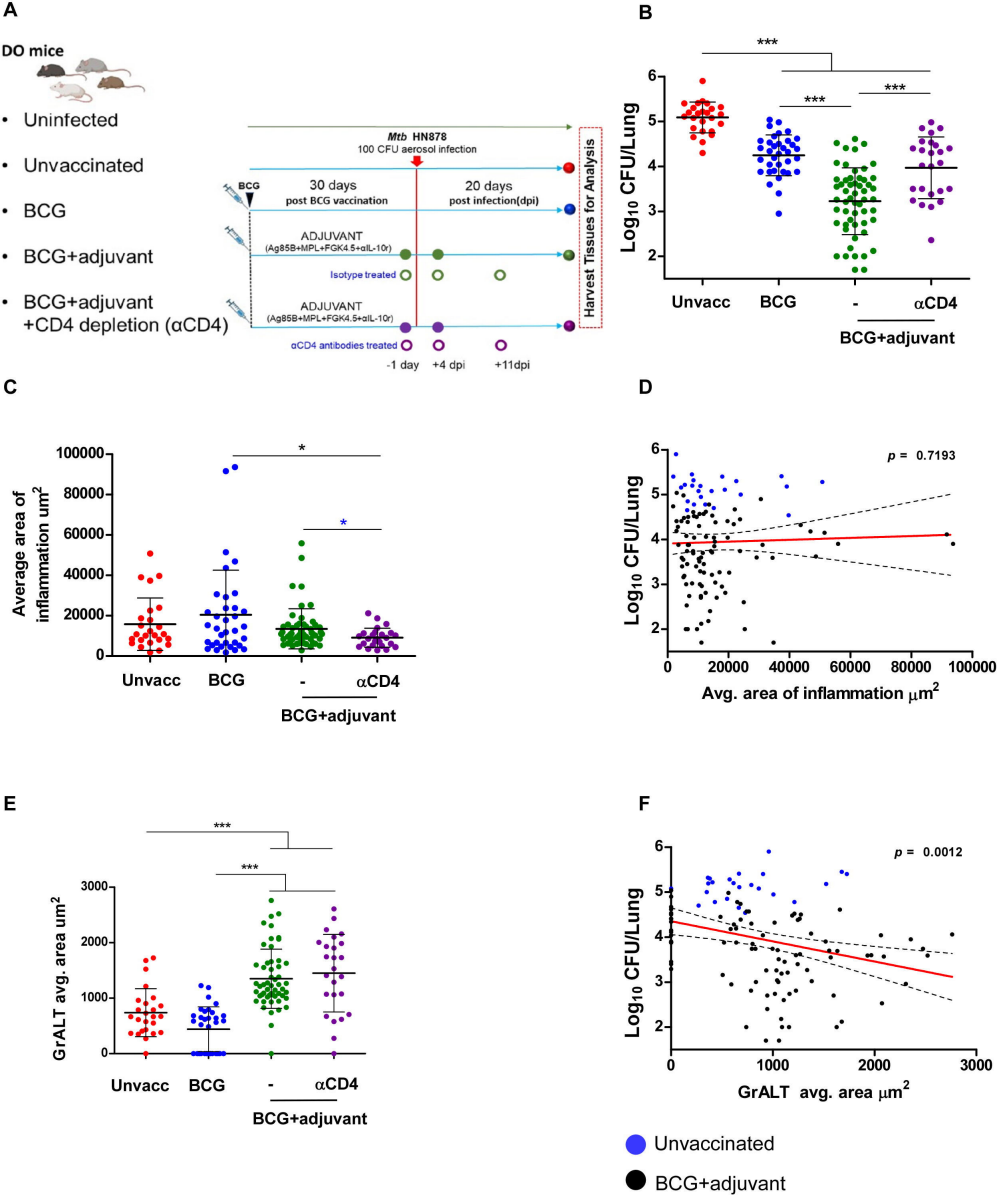
Targeting innate lung pathway in BCG-vaccinated mice at the time of *Mtb* infection enhances *Mtb* control in a CD4-dependent manner. (**A**) Individual DO mice were vaccinated with s.c. BCG (BCG—blue circles) rested for 30 days, following which they received adjuvant (MPL + FGK4.2 + α-IL-10 + Ag85B) −1 and 4 days post-infection (BCG + adjuvant—green circles). Unvaccinated mice were also included as controls (red circles). The mice were then challenged with *Mtb* HN878 (100 CFU). In one group, BCG + adjuvant *Mtb*-infected mice received three doses of CD4 depletion antibody 1 day before infection and weekly on day 4 and day 11 following infection (300 μg/mouse), (BCG + adjuvant CD4 depleted—purple circles). (**B**) Lung *Mtb* CFU was determined in lung homogenates from groups described by plating at 20 dpi. (**C**) FFPE lung sections were subjected to H&E staining, and lung inflammation was quantified, and the average area of inflammation was quantified for each individual DO mouse. (**D**) Linear correlation analysis of lung inflammation versus log_10_ *Mtb* CFU was calculated and blue circles (Unvaccinated) and black circles (BCG + adjuvant) were performed using Pearson’s correlation on GraphPad Prism. (**E**) FFPE lung sections were also analyzed by immunofluorescence using antibodies specific for B220, and the total area occupied by B-cell follicles per lobe was quantified using the morphometric tool of the Zeiss Axioplan (Carl Zeiss, Oberkochen, Germany), and the average area occupied by GrALT is included. (**F**). Linear correlation analysis of average GrALT area versus log_10_ *Mtb* CFU was calculated, and blue circles (Unvaccinated) and black circles (BCG+adjuvant) were performed using Pearson correlation on GraphPad Prism. Statistical significance was verified by one-way ANOVA with Tukey’s correction or (shown with blue asterisk) by unpaired two-tailed Students *t*-test, *n* = 25–56 individual DO mice. All data are mean ± s.d. *P ≤ 0.05; **P ≤ 0.01; ***P ≤ 0.001; ****P ≤ 0.0001.

Previous studies from our group and others (2–5) have demonstrated that the DO mouse model of TB is a valuable model to help delineate immune features associated with TB disease or protection. Thus, we next addressed whether BCG vaccination induced protection upon challenge with a hypervirulent clinical strain of *Mtb* HN878 in DO BCG-vaccinated mice. Individual DO mice were subcutaneously (s.c.) vaccinated with BCG, rested for 30 days, following which mice were infected with low-dose *Mtb* HN878 infection. Unvaccinated (Unvacc) mice were also infected with *Mtb* HN878. Additionally, to activate the innate lung pathways and accelerate initiation of adaptive immune responses, adjuvants targeting TLR4 and CD40 pathways were delivered mucosally along with *Mtb* antigen (Ag85B) on days −1 and +4 following infection to BCG-vaccinated DO mice (TLR4 agonist—MPL, along with CD40 agonist, FGK4.5); IL-10-neutralizing antibody was also delivered systemically—henceforth referred to as BCG + adjuvant (8). Finally, to assess the direct role of CD4^+^ T cells in mediating vaccine-induced protection, a group of BCG + adjuvant-vaccinated DO mice following *Mtb* infection also received CD4^+^-depleting antibody (BCG + adjuvant + CD4 depletion [αCD4]). The experimental scheme is displayed in Fig. 1A.

All groups were sacrificed at 20 days post-infection (dpi), as at this time point vaccinated mice have mounted a significant CD4^+^ T-cell response, but unvaccinated *Mtb*-infected mice have not yet generated optimal CD4^+^ T-cell responses (9). As before (2), *Mtb* infection in DO mice resulted in a heterogeneous response to infection in individual DO mice with variable log_10_ *Mtb* lung CFU ranging between 4 and 6, with a median CFU of 5.16. BCG vaccination in DO mice prior to *Mtb* infection induced significant reduction of *Mtb* CFU with a median log_10_ *Mtb* lung CFU of 4.32 (Fig. 1B). Importantly, when DO BCG-vaccinated mice also received adjuvant treatment, they exhibited superior protection when compared with BCG vaccination alone, with lung *Mtb* CFU in some mice at the level of detection, and median log_10_ *Mtb* lung CFU dropped to 3.27 (Fig. 1B). This improved protection in BCG + adjuvant-vaccinated DO mice was CD4^+^ T cell dependent, as protection was reversed when mice also received CD4^+^-depleting antibody with a median log_10_ *Mtb* lung CFU of 4.08 (Fig. 1B). Thus, our results show that BCG vaccination in DO mice induced protection which could further be improved by targeting lung innate pathways, and that this enhanced vaccine-induced protection was CD4^+^ T cell dependent.

We then measured lung inflammatory areas in the four groups of DO mice and found that while *Mtb* infection at this early time point by itself induced lung inflammatory foci, the inflammation was comparable in BCG-vaccinated and BCG + adjuvant-vaccinated *Mtb*-infected groups, when compared with unvaccinated *Mtb*-infected lungs (Fig. 1C). Accordingly, while we did not find any correlation between the *Mtb* CFU and average area of lung inflammation (Fig. 1D), we found that CD4^+^ T-cell depletion in the BCG + adjuvant group reversed the lung inflammation significantly when compared with both BCG-treated and BCG + adjuvant-treated groups (Fig. 1C). Our previous studies have associated the generation of TB granulomas that harbor B-cell lymphoid-like structures (granuloma-associated lymphoid tissue [GrALT]) with the control of *Mtb* infection in DO mice (2, 4, 10). Accordingly, we found that individual DO mice which were *Mtb*-infected exhibited the formation of GrALT within the lung (Fig. 1E). However, BCG vaccination did not further improve the formation of GrALT within the lung (Fig. 1E). Activation of lung innate pathways resulted in significantly increased GrALT in the BCG + adjuvant group, and this response was not reversed when CD4^+^ T cells were depleted (Fig. 1E). Indeed, when we carried out linear correlation analysis between *Mtb* CFU and GrALT formation, we found a significant correlation between increased GrALT induced in vaccinated DO mice and decreased lung *Mtb* CFU (Fig. 1F). Thus, our results effectively show that BCG vaccination and targeting the lung innate pathways in DO mice did not enhance overall inflammation but induced significant formation of GrALT within the granulomas and correlated with better *Mtb* control.

### Activation of lung innate pathways in BCG-vaccinated DO mice enhanced the accumulation of lung IL-17-producing CD4^+^ T cells

**Fig 2:**
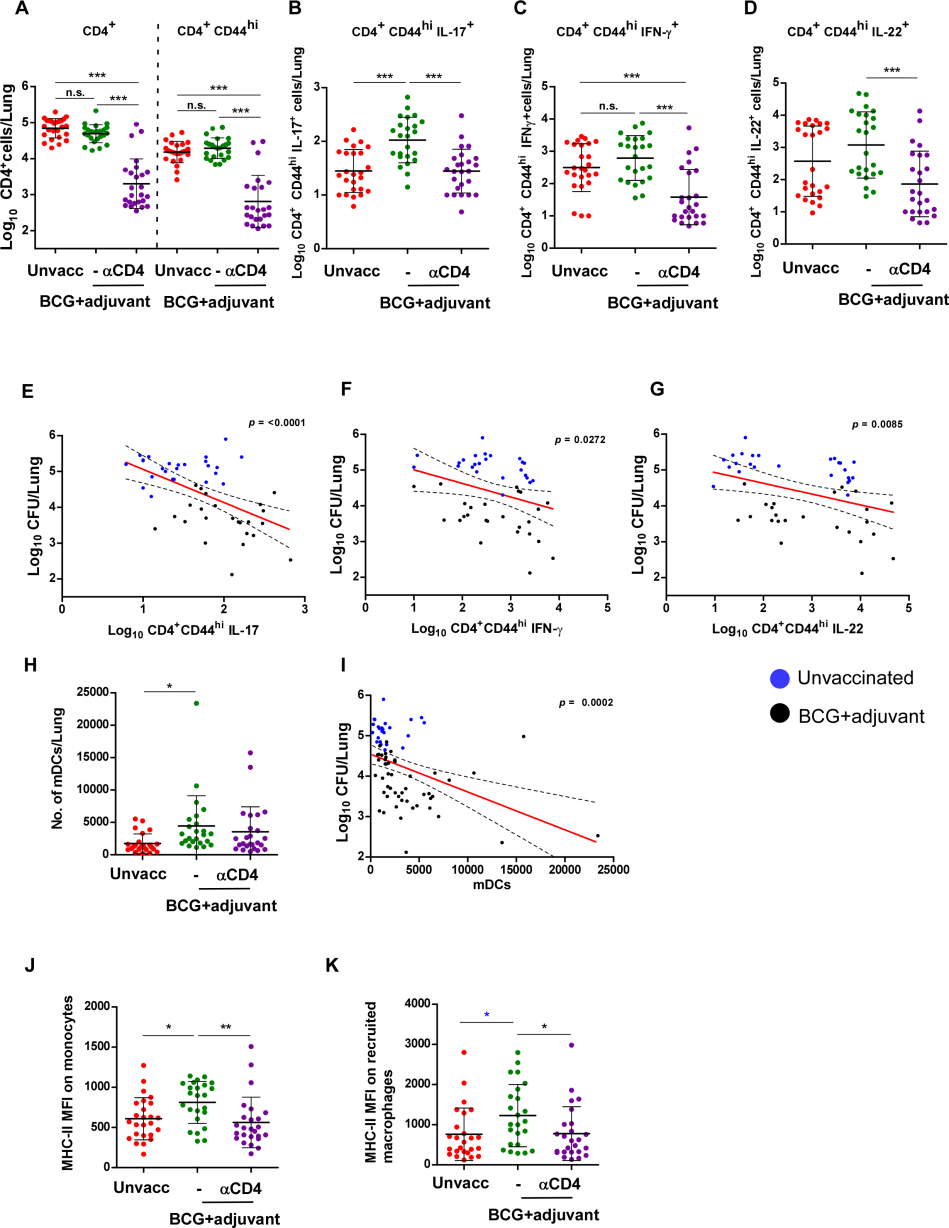
Targeting the lung innate pathway activates Th17 responses and enhances GrALT to improve *Mtb* control. Individual DO mice were left unvaccinated, vaccinated with BCG, rested for 30 days, and challenged with *Mtb* HN878 (100 CFU), and received adjuvant as described in Fig. 1 (BCG + adjuvant). One group of BCG + adjuvant *Mtb*-infected mice received CD4 depletion antibodies following infection (BCG + adjuvant CD4 depleted). (**A**) Using flow cytometry, total CD4^+^ and CD4^+^ CD44^hi^ responses were measured in single-cell lung suspensions and (**B**) IFN-γ-, (**C**) IL-17-, and (**D**) IL-22-producing CD4^+^CD44^hi^ cells were enumerated using intracellular staining and flow cytometry. Linear correlation analysis of (**E**) log_10_ CD4^+^CD44^hi^ IFN-γ-producing cells, (**F**) log_10_ CD4^+^CD44^hi^ IL-17-producing cells, and (**G**) log_10_ CD4^+^CD44^hi^ IL22-producing cells versus log_10_ *Mtb* CFU was calculated; blue circles (Unvaccinated) and black circles (BCG + adjuvant) were carried out using Pearson’s correlation on GraphPad Prism. (**H**) The number of lung myeloid DCs (mDCs) was measured in lung suspensions using flow cytometry and gated as described in Materials and Methods. (**I**) Linear correlation analysis of number of mDCs versus log_10_ *Mtb* CFU was calculated, blue circles (Unvaccinated) and black circles (BCG + adjuvant) were performed using Pearson’s correlation on GraphPad Prism. The MHC-II mean fluorescence intensity (MFI) on (**J**) lung monocytes and (**K**) recruited macrophages was measured. Statistical significance was verified by one-way ANOVA with Tukey’s correction; *n* = 25–56 biological replicates for all figures. An unpaired two-tailed Student’s *t*-test was carried out only to compare the two groups (blue asterisk), unvaccinated and BCG + adjuvant-vaccinated mice in panel **K**). All data are mean ± s.d. *P ≤ 0.05; **P ≤ 0.01; ***P ≤ 0.001; ****P ≤ 0.0001. n.s., not significant.

As activation of lung innate pathways in BCG + adjuvant-vaccinated hosts provided significantly better lung *Mtb* control, we evaluated the CD4^+^ T-cell responses in this group and compared it to the unvaccinated *Mtb*-infected group. While total activated CD4^+^ CD44^hi^ cells, IFN-γ-producing and IL-22-producing activated CD4^+^ T cells were not significantly different in unvaccinated *Mtb*-infected lungs and BCG + adjuvant *Mtb*-infected lungs, the number of lung IL-17-producing CD4^+^ CD44^hi^ T cells was higher in the BCG + adjuvant *Mtb*-infected group when compared with unvaccinated *Mtb*-infected lungs (Fig. 2A through D). Indeed, CD4 depletion in the BCG-vaccinated + adjuvant group resulted in the loss of activated CD4^+^CD44^hi^ cells, as well as loss of all cytokine-producing CD4^+^CD44^hi^ T cells, including those producing IL-17, IFN-γ, and IL-22 (Fig. 2B through D). While assessing whether cytokine-producing CD4^+^CD44^hi^ cell accumulation correlated with improved *Mtb* CFU, we found that IL-17-producing CD4^+^CD44^hi^ cells correlated with lower *Mtb* CFU to a higher extent, while IFN-γ-producing and IL-22-producing CD4^+^CD44^hi^ T cells also showed a positive correlation with decreased lung *Mtb* CFU but to a smaller extent than IL-17-producing CD4^+^ T cells (Fig. 2E through G). Multiple linear regression, including IL-17, IFN-γ, and IL-22, confirmed that IL-17 was the strongest independent correlate of lower bacterial burden (regression coefficient β = −0.916, *P* = 1.9 × 10^−4^, two-sided *t* test compared to β = 0), with a smaller contribution from IL-22 (β = −0.455, *P* = 0.028), and no significance to IFN-γ in the joint model (β = 0.449, *P* = 0.156). To address potential collinearity, we computed variance inflation factors (VIFs) for the joint model, which were all within acceptable limits (<5; IL-17 = 1.19, IFN-γ = 4.76, IL-22 = 4.40). However, we noted a high correlation between IFN-γ and IL-22 (Pearson *r* = 0.88). Likelihood ratio tests indicated that adding IFN-γ to the IL-17 model did not improve fit (likelihood-ratio test *P* = 0.31), whereas adding IL-22 did (*P* = 0.049). Together, these analyses indicate that IL-17 remained the most statistically significant independent correlate of reduced bacterial burden, with a modest contribution from IL-22 and no independent effect of IFN-γ. These results suggest that CD4^+^ T cells, particularly IL-17-producing CD4^+^CD44^hi^ cells accumulating in BCG + adjuvant lungs, may play a role in mediating enhanced *Mtb* control seen in this group.

Additionally, while the total number of lung monocytes and recruited macrophages (RMs) was not different between the three groups (Fig. S1A and B), we found that the accumulation of lung mDCs was increased in the lungs of BCG + adjuvant-vaccinated mice compared with unvaccinated lungs and correlated significantly with decreased *Mtb* CFU (Fig. 2H and I). Activation of lung innate responses was evident with upregulation of MHC Class II expression on monocytes and RMs in the BCG + adjuvant group when compared with unvaccinated mice (Fig. 2J and K). Furthermore, CD4^+^ T-cell depletion resulted in decreased expression of Class II on both monocytes and RMs, suggesting that the treatment with adjuvants was not directly responsible for the activation of these innate cells; accumulation of mDCs and increased T-cell responses were likely mediating increased activation of monocytes and RMs in the lung.

### BCG vaccination-induced protection in DO mice is associated with pathways involved in lung repair and the TLR10 cascade

**Fig 3:**
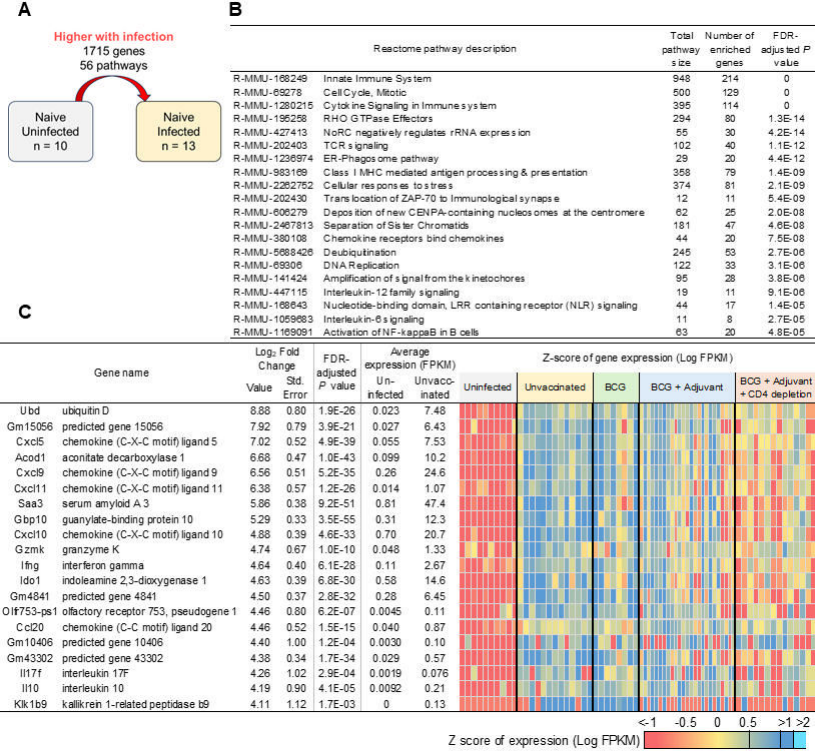
Early genes and pathways that are upregulated in lungs of *Mtb*-infected DO mice reflect activation of innate immune and cytokine pathways. (**A**) Representation of differential gene expression comparisons of interest, with the current comparison highlighted. RNA was extracted from lungs of individual unvaccinated *Mtb*-infected mice at 20 dpi, and differentially expressed genes and pathways that were upregulated when compared with naïve uninfected lungs were determined. Lung transcriptional profiles from naïve uninfected RNA were used from reference 2. (**B**) The top 20 most significantly enriched Reactome pathways and (**C**) the top 20 genes (ranked by fold change) that were upregulated in *Mtb*-infected lungs over naïve uninfected lungs are shown. Differential gene expression statistics are from DESeq2 output, and heatmaps represent expression levels in each sample.

Our published data in the lungs of DO *Mtb*-infected mice at later time points (50 dpi) showed enrichment of transcriptional signatures associated with the innate immune system, immune system, and cytokine signaling pathways (2). Accordingly, we isolated RNA from individual DO *Mtb*-infected mice at early time points to further delineate the early lung transcriptional profiles following *Mtb* infection without vaccination (“unvaccinated”; *n* = 13), under conditions of prior BCG vaccination before *Mtb* infection (“BCG”; *n* = 8), and in lungs where BCG vaccinated along with innate pathway activation resulted in enhanced *Mtb* control (“BCG + adjuvant,” *n* = 14; Fig. 3A; All sample metadata and accessions provided in Table S1). Differentially expressed genes between sample sets as described below were performed using DESeq2 (11) (*P* ≤ 0.01), including uninfected samples collected from the previous study (2) (*n* = 10), and functional enrichment of differentially expressed genes performed based on Reactome pathways (12). All gene expression and differential expression for all genes in all comparisons are provided in Table S2.

Among the 1,715 differentially expressed genes in the lungs of individual DO *Mtb*-infected mice at 20 dpi relative to uninfected mice, we found significant enrichment of pathways associated with innate immune system, TCR signaling, cellular responses to stress, and cytokine signaling in immune response among others (Fig. 3B). Incidentally, the top most significantly upregulated genes included IFN-γ, IFN-inducible chemokines such as *CXCL9*, *CXCL10,* and *CXCL11*, Gbp family proteins including *Gbp10*, immunosuppressive molecules such as *Ido1* and *Il10*, as well as serum amyloid A3 (Fig. 3C; Table S3A). Additionally, when the differential gene expression of the 13 mouse orthologs of the 16 ACS signature genes identified in Zak et al*.* (13) was compared between the *Mtb* infected and uninfected controls, 13 of the 16 genes were induced at this early time point (Table S4). These results confirm that even at this early time point, lungs of individual DO *Mtb-*infected mice respond to infection by inducing an innate immune signature of activation.

**Fig 4:**
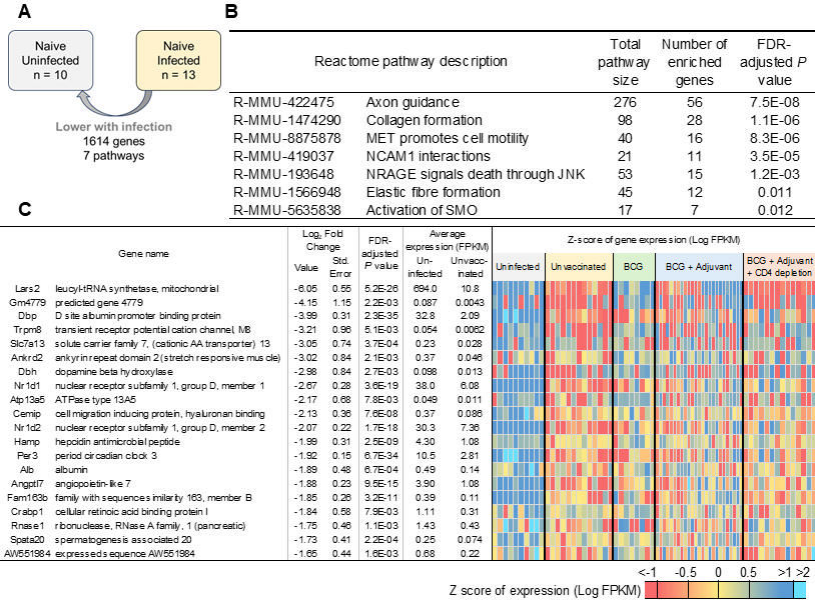
Early genes and pathways that are downregulated in lungs of *Mtb*-infected DO mice reflect a reduction in lung repair pathways. (**A**) Representation of differential gene expression comparisons of interest, with the current comparison highlighted. RNA was extracted from lungs of individual unvaccinated *Mtb*-infected mice at 20 dpi, and differentially expressed genes and pathways that were downregulated when compared with naïve uninfected lungs were determined. Lung transcriptional profiles from naïve uninfected RNA were used from reference 2. (**B**) All seven significantly enriched Reactome pathways and (**C**) the top 20 genes (ranked by fold change) that were downregulated in *Mtb*-infected lungs over naïve uninfected lungs are shown. Differential gene expression statistics are from DESeq2 output, and heatmaps represent expression levels in each sample.

In contrast, of the 1,614 genes downregulated in the lungs of *Mtb*-infected DO mice compared to naïve uninfected lung, we identified significant enrichment for Reactome pathways including collagen formation, elastic fiber formation, MET promotes cell motility, axon guidance, and NCAM1 interactions, suggesting that genes involved in lung repair functions are downregulated early during *Mtb* infection (Fig. 4A and B; Table S3B). The top most significantly downregulated genes included *Lars2,* which encodes a class 1 aminoacyl-tRNA synthetase, and mitochondrial leucyl-tRNA synthetase, which has been shown to play a role in metabolism of proteins (14) (Fig. 4C). Studies have described the association of circadian molecules in club cells, alveolar macrophages, and fibroblasts, in response to lung injury and inflammatory mediators (15). Accordingly, we found several clock-associated molecules such as D site albumin promoter binding protein (*Dbp*), nuclear receptor subfamily 1, group D, member 1 (16, 17) and member 2 (18) (*Nrd1* and *Nrd2*), and period circadian clock 3 (Per3) (19). These results suggest that early following infection, while inflammatory mediators are induced, genes associated with collagen formation, clock genes, and lung repair genes are downregulated during *Mtb* infection.

We additionally compared DO mice that were BCG-vaccinated and *Mtb*-infected to uninfected mice, identifying 2,229 overexpressed genes in the lung, including 1,156 genes also upregulated in the unvaccinated *Mtb*-infected mice compared to uninfected. These overlapping 1,156 genes were enriched for pathways including induction of inflammatory cytokines (Table S5), suggesting that an activated lung landscape in *Mtb*-infected BCG-vaccinated mice is similar to *Mtb*-infected lungs.

**Fig 5:**
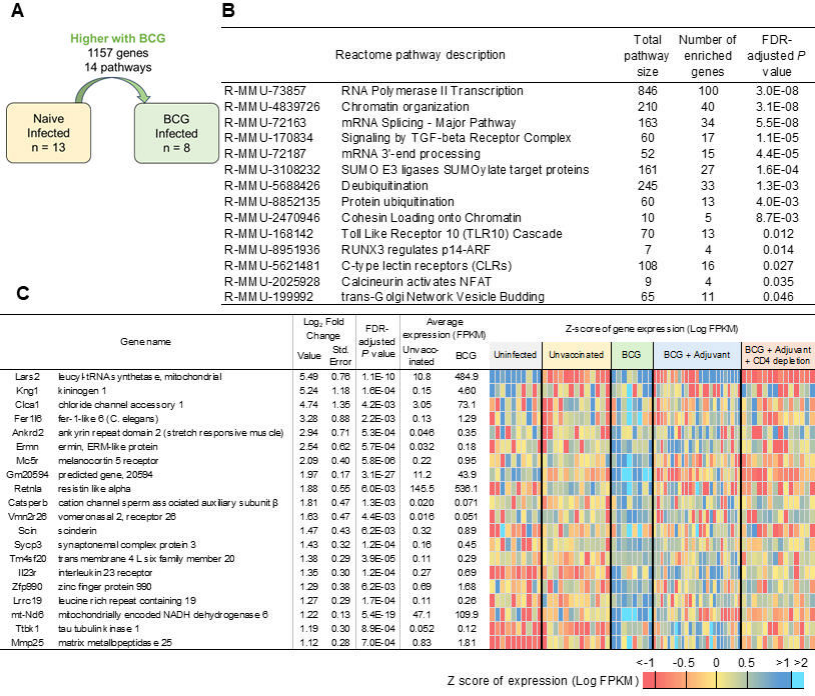
Genes and pathways that are upregulated uniquely in lungs of BCG-vaccinated *Mtb*-infected DO mice, which reflect activation of TGF-β signaling and lung repair. (**A**) Representation of differential gene expression comparisons of interest, with the current comparison highlighted. RNA was extracted from lungs of individual BCG-vaccinated *Mtb*-infected mice at 20 dpi, and differentially expressed genes and pathways that were upregulated when compared with *Mtb*-infected lungs were determined. (**B**) All 14 significantly enriched Reactome pathways and (**C**) the top 20 genes (ranked by fold change) that were upregulated in BCG-vaccinated *Mtb*-infected lungs over *Mtb*-infected lungs are shown. Differential gene expression statistics are from DESeq2 output, and heatmaps represent expression levels in each sample.

We then focused on the 1,157 genes expressed higher and 994 genes that were expressed lower in BCG-vaccinated *Mtb-*infected lungs when compared with *Mtb*-infected lungs (Fig. 5A; Table S3C). The pathways that were induced higher in BCG-vaccinated *Mtb*-infected DO mice included pathways associated with TGF-β receptor complex, chromatin organization, RNA polymerase II transcription, and TLR10 cascade (Fig. 5B). Interestingly, *Lars2,* which is downregulated during *Mtb* infection, was the highest induced gene in the context of BCG vaccination and *Mtb* infection (Fig. 5C). *Lars2* expression in B cells has been associated with TGF-β regulatory mechanism in mouse and human progressive colorectal cancer (20). *Lars2*-expressing B cells also exhibited a leucine nutrient preference and displayed active mitochondrial aminoacyl-tRNA biosynthesis and programmed mitochondrial nicotinamide adenine dinucleotide (NAD^+^) regeneration and oxidative metabolism (20). TLR10 ligands, such as lipopolysaccharide, synthetic diacylated lipoprotein (FSL-1), and flagellin, can inhibit as well as activate downstream pathways through MyD88- and TRIF-dependent signaling pathways. It may regulate MAPK/NF-κB and PI3K/Akt signaling pathways, which regulate the innate immune responses and differentiation of primary human monocytes into effective dendritic cells (21, 22). Kininogen is a protein that is part of the kallikrein-kinin system, a complex involved in inflammation, blood coagulation, and vascular permeability and in this case likely driving lung repair (23, 24). Similarly, *Retnla*, also known as *Fizz1*, plays a role in lung repair and fibrosis (25). *Clca1* (chloride channel accessory protein) plays a role in mucus secretion and lung repair and is among the top genes induced in the BCG-vaccinated lung. Together, our data suggest that in BCG-vaccinated DO mice, the induction of inflammatory genes overlapping significantly with *Mtb* infection (Table S5A) is countered by the induction of genes involved in lung repair (Fig. 5). This is in stark contrast to the same genes that are downregulated in the context of *Mtb* infection.

### BCG vaccination in DO mice downregulated pathways linked to metabolism, respiration, and cell cycle

**Fig 6:**
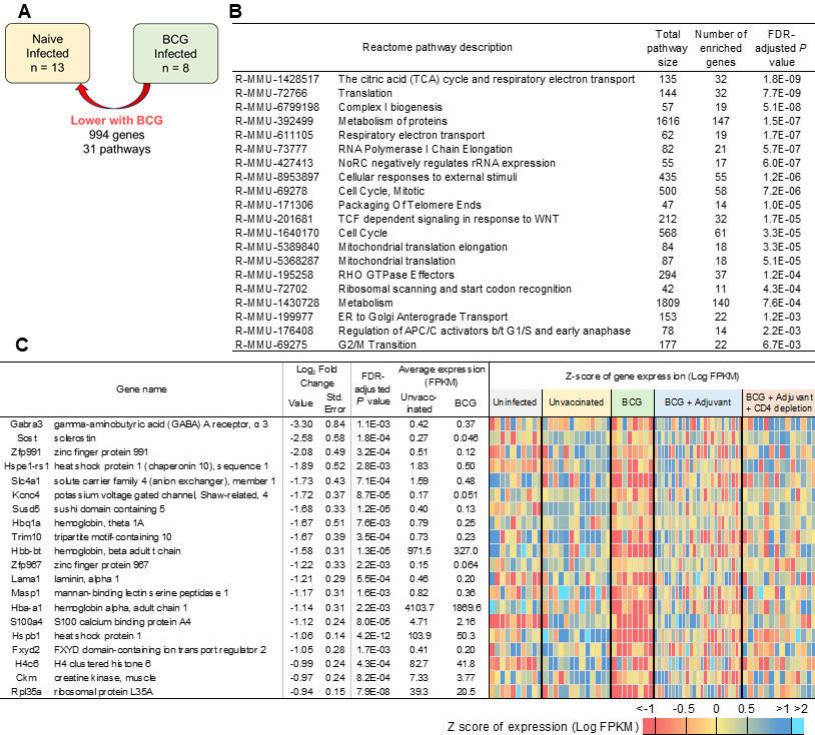
Genes and pathways that are downregulated in lungs of BCG-vaccinated *Mtb*-infected DO mice reflect reduction in TCA cycle and electron transport. (**A**) Representation of differential gene expression comparisons of interest, with the current comparison highlighted. RNA was extracted from lungs of individual BCG-vaccinated *Mtb*-infected mice at 20 dpi, and differentially expressed genes and pathways that were downregulated when compared with *Mtb*-infected lungs were determined. (**B**) The top 20 most significantly enriched Reactome pathways and (**C**) the top 20 genes (ranked by fold change) that were downregulated in BCG-vaccinated *Mtb*-infected lungs over *Mtb*-infected lungs are shown. Differential gene expression statistics are from DESeq2 output, and heatmaps represent expression levels in each sample.

The 994 genes that were downregulated in the lungs of BCG-vaccinated DO mice relative to uninfected mice were significantly enriched for pathways involved in metabolism of proteins, TCA cycle and respiratory electron transport, and cell cycle (Fig. 6A and B; Table S3D). For example, the gene sclerostin, *Sost,* has a potential role in altering homeostasis of endothelial and smooth muscle cells of the pulmonary vessel wall (26) (Fig. 6C). The solute carrier family 4 (*Slc4a14*) is an important protein responsible for the transport of various ions across the cell membrane and mediating diverse physiological functions, such as the ion transporting function, protein-to-protein interactions, and molecular transduction (27). Laminin alpha 1 (*lama1*) is a genetic modifier of TGF-β-induced pulmonary fibrosis (28). In addition, *Susd5,* which is regulated by TGF-β, was also downregulated (29). Thus, although lung repair genes are induced in BCG-vaccinated lungs, there are still pathways that are further downregulated in the BCG-vaccinated lungs that regulate respiration and cell cycle.

### The BCG + adjuvant DO mice group showed enhanced protection against *Mtb* infection due to lung innate pathway activation and T- and B-cell accumulation

**Fig 7:**
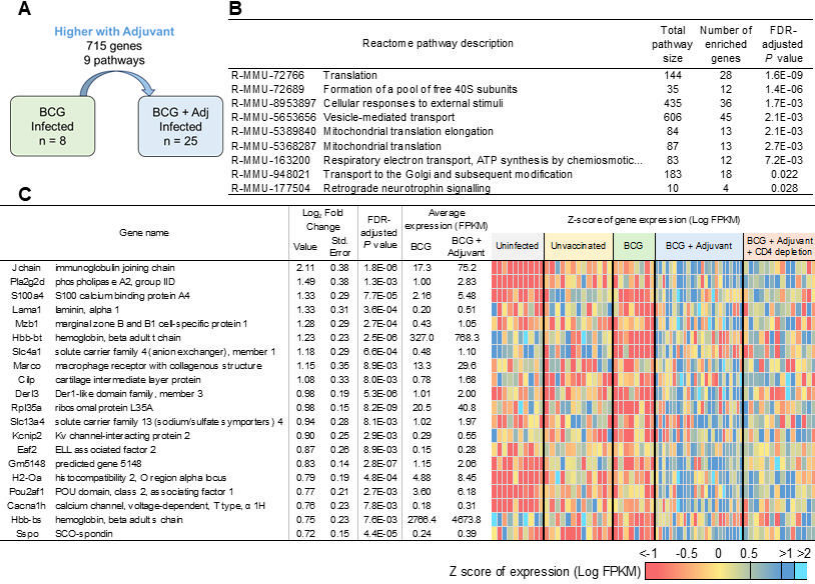
Genes and pathways that are upregulated uniquely in lungs of BCG + adjuvant-vaccinated *Mtb*-infected DO mice, which reflect cellular responses to external stimuli. (**A**) Representation of differential gene expression comparisons of interest, with the current comparison highlighted. RNA was extracted from lungs of individual BCG + adjuvant-vaccinated *Mtb*-infected mice at 20 dpi, and differentially expressed genes and pathways that were upregulated when compared with BCG-vaccinated *Mtb*-infected lungs were determined. (**B**) All nine significantly enriched Reactome pathways and (**C**) the top 20 genes (ranked by fold change) that were upregulated in BCG + adjuvant-vaccinated *Mtb*-infected lungs over BCG-vaccinated *Mtb*-infected lungs are shown. Differential gene expression statistics are from DESeq2 output, and heatmaps represent expression levels in each sample.

Since the activation of lung innate pathways in the BCG + adjuvant DO mouse group showed significantly enhanced protection upon *Mtb* infection, we compared transcriptional profiles in the lungs of this group to the lungs of BCG-vaccinated *Mtb*-infected lungs (Fig. 7A). Approximately 62% of the genes that were higher in BCG compared to the unvaccinated cohort were also higher in BCG compared to the BCG+adjuvant cohort, and these overlapping genes were significantly enriched for mRNA splicing and the adaptive immune system (Table S5). Of the genes that are uniquely upregulated in BCG + adjuvant *Mtb*-infected lungs compared with BCG-vaccinated *Mtb*-infected lung, there were 715 genes and 9 pathways associated with translation, cellular responses to external stimuli, and mitochondrial translation (Fig. 7B; Table S3E). The top gene induced in the lungs of this group was *Jchain* (immunoglobulin joining chain), which is known to link two monomer units of either IgM or IgA and is associated with lymphoid follicles (30). Furthermore, the other top induced gene of interest, MZB1, is marginal zone B and B1 cell-specific protein 1, which promotes J-chain binding to IgA (31) and is also involved in regulating inflammation. In addition, *S100A4* is known to regulate Lck/Fyn activity in CD5 signaling to promote RORγt expression and Th17 polarization (32). These transcriptional profiles fit well with the induction of both Th17 cells as well as the formation of GrALT that is observed in the lungs of the BCG + adjuvant *Mtb*-infected DO mice (Fig. 1E and 2C).

**Fig 8:**
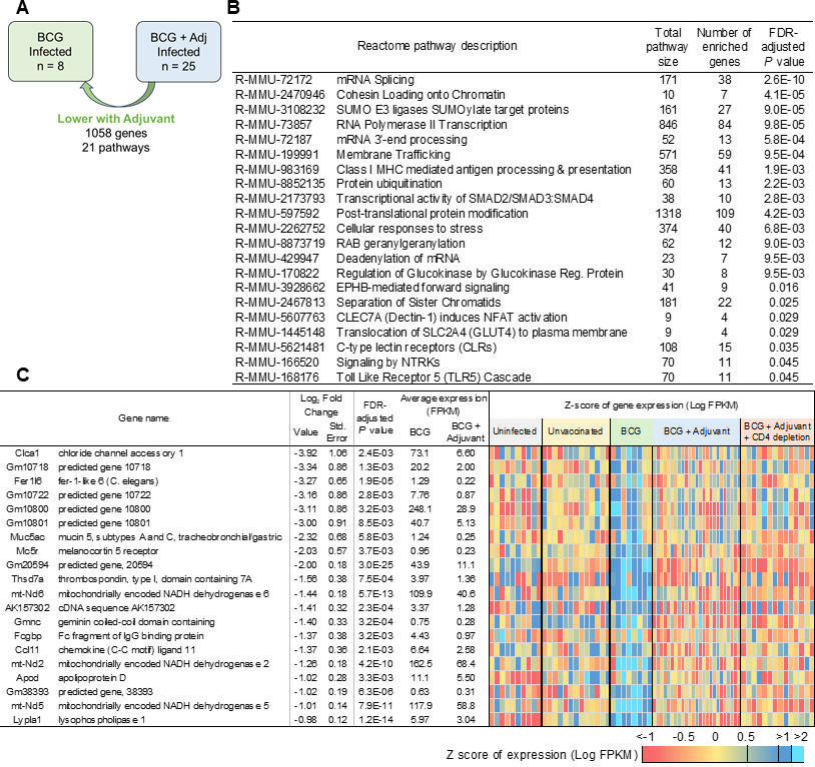
Genes and pathways that are downregulated in lungs of BCG + adjuvant-vaccinated *Mtb*-infected DO mice reflect reduction in mRNA splicing. (**A**) Representation of differential gene expression comparisons of interest, with the current comparison highlighted. RNA was extracted from lungs of individual BCG + adjuvant-vaccinated *Mtb*-infected mice at 20 dpi, and differentially expressed genes and pathways that were downregulated when compared with BCG-vaccinated *Mtb*-infected lungs were determined. (**B**) All 21 significantly enriched Reactome pathways and (**C**) the top 20 genes (ranked by fold change) that were downregulated in BCG + adjuvant-vaccinated *Mtb*-infected lungs over BCG-vaccinated *Mtb*-infected lungs are shown. Differential gene expression statistics are from DESeq2 output, and heatmaps represent expression levels in each sample.

In the context of gene expression in BCG + adjuvant *Mtb-*infected lungs compared with BCG-vaccinated *Mtb*-infected lungs, 1,058 genes were downregulated and involved pathways associated with mRNA splicing, mRNA 3′-end processing, protein ubiquitination, and post-translation modification (Fig. 8A and B; Table S3E). The list of top 20 downregulated genes includes *Clca1*, *Muc5ac mucin 5*, *subtypes A* and *C*, *Fcgbp* (Fig. 8C). *Clca1* regulates airway mucus production and ion secretion through the calcium-activated ion channel Tmem16A (33). Airway mucus is a hydrophilic gel made up of mucins, proteins, salt, and water. It traps and clears inhaled pathogens, protecting the lungs. Two mucins, *Muc5ac* and *Muc5b*, are expressed in the lungs, with Muc5b being primarily protective and MUC5ac being pathologic due to increased expression in disease states (34). Similarly, IgGFc-binding protein (*Fcgbp*) is a mucin that was first detected in the intestinal epithelium. It plays an important role in innate mucosal epithelial defense, tumor metastasis, and tumor immunity (35). These results suggest that improved protection afforded by activation of lung innate immunity in the BCG + adjuvant group limits the production of mucin and associated pathways.

### CD4^+^ T-cell depletion in BCG + adjuvant-vaccinated DO mice resulted in compromised immune functionality

**Fig 9:**
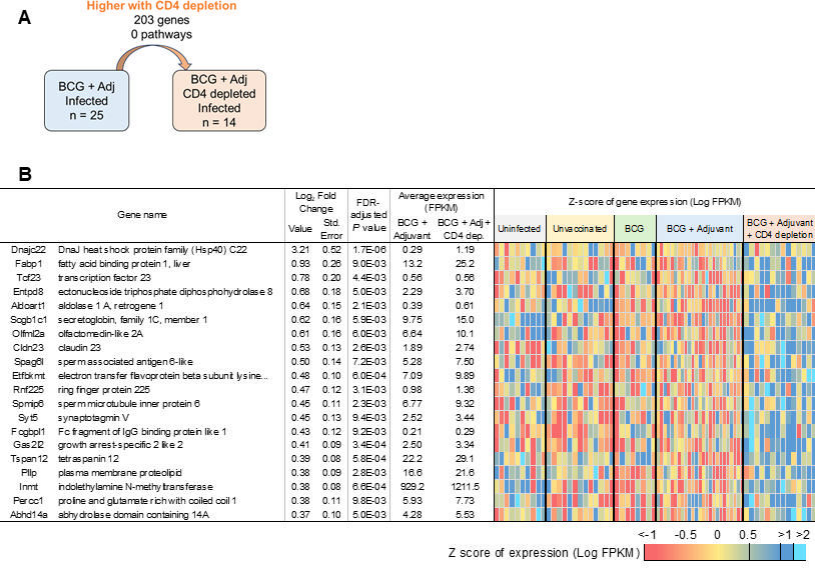
CD4 T-cell depletion in BCG + adjuvant-vaccinated *Mtb*-infected DO mice induces heat shock protein genes. (**A**) Representation of differential gene expression comparisons of interest, with the current comparison highlighted. RNA was extracted from lungs of individual BCG + adjuvant-vaccinated *Mtb*-infected mice, where CD4 depletion was carried out, and at 20 dpi, differentially expressed genes that were upregulated when compared with BCG + adjuvant-vaccinated *Mtb*-infected lungs were determined. (**B**) The top 20 genes (ranked by fold change) that were upregulated in BCG + adjuvant-vaccinated *Mtb*-infected lungs with CD4 T-cell depletion over BCG + adjuvant-vaccinated *Mtb*-infected lungs are shown. Differential gene expression statistics are from DESeq2 output, and heatmaps represent expression levels in each sample.

**Fig 10:**
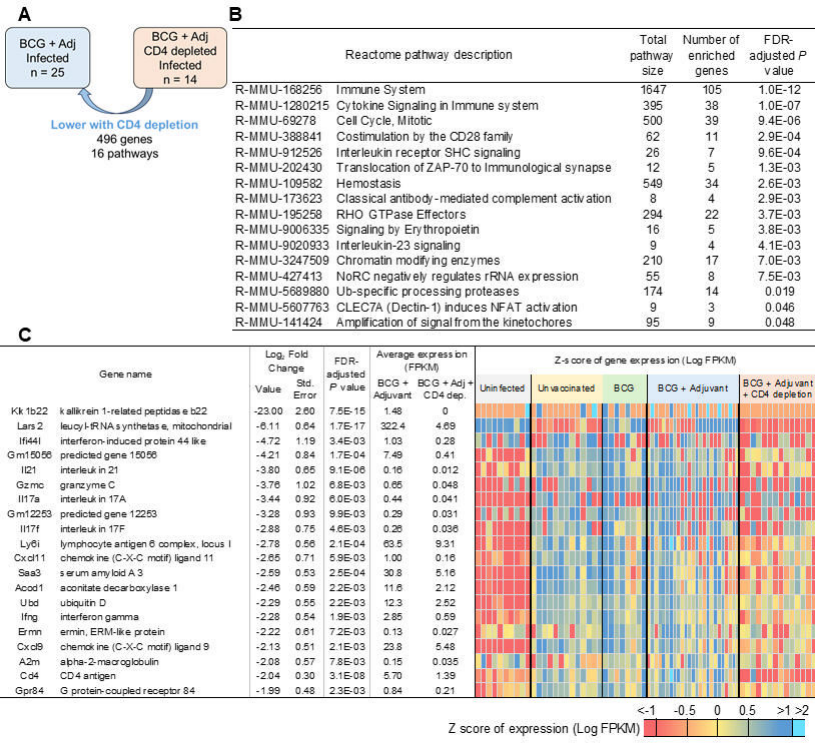
CD4 T-cell depletion in BCG + adjuvant-vaccinated *Mtb*-infected DO mice downregulates immune and cytokine signaling cascades. (**A**) Representation of differential gene expression comparisons of interest, with the current comparison highlighted. RNA was extracted from lungs of individual BCG + adjuvant-vaccinated *Mtb*-infected mice, where CD4 depletion was carried out, and at 20 dpi, differentially expressed genes that were downregulated when compared with BCG + adjuvant-vaccinated *Mtb*-infected lungs were determined. (**B**) All 16 significantly enriched Reactome pathways and (**C**) the top 20 genes (ranked by fold change) that were downregulated in BCG + adjuvant-vaccinated *Mtb*-infected lungs with CD4^+^ T-cell depletion over BCG + adjuvant-vaccinated *Mtb*-infected lungs are shown. Differential gene expression statistics are from DESeq2 output, and heatmaps represent expression levels in each sample.

Upon depletion of CD4^+^ T cells in the BCG + adjuvant group, we found that only 203 genes were higher when compared with mice that received BCG + adjuvant and isotype antibody (Fig. 9A). No Reactome pathways were significantly enriched in the lung, and the only gene that was induced over twofold in the lungs was DnaJ heat shock protein family (*Hsp40*) member C22 (*Dnajc22*) (36), that functions as a co-chaperone, potentially involved in protein folding, unfolded protein binding, and protein-folding chaperone binding (Fig. 9B). In sharp contrast, when CD4^+^ T cells were depleted, we found that 496 genes and 16 pathways were expressed at lower levels in lungs of BCG + adjuvant-treated mice such as those involved in immune system, cytokine signaling in immune system, IL-23 signaling and costimulation by the CD28 family (Fig. 10A and B; Table S3G). The top gene that was reduced upon CD4 T-cell depletion in BCG-vaccinated + adjuvant was kallikrein 1-related peptidase b22 (*Klk1b22*), which enables peptidase activity and is known to regulate systemic metabolism (37) (Fig. 10C). Incidentally, the second most reduced gene expression that was reduced in CD4-depleted BCG-vaccinated + adjuvant was *Lars2*. Other important cytokines that were reduced in this group were relevant cytokines such as *Il17a*, *Il17f*, *Ifng*, and *Il21*. Additionally, CD4 antigen expression was also reduced, confirming the depletion of CD4 T cells in the BCG-vaccinated + adjuvant group. Both *Cxcl9* and *Cxcl11* levels were also reduced in this group, as was the inflammatory marker *Saa3* (Fig. 10B). Together, the transcriptional profile in CD4^+^ T-cell-depleted BCG-vaccinated + adjuvant group demonstrated a clear lack of immune signaling, cytokine production, and reduction in chemokines, resulting in a compromised immune functionality.

## DISCUSSION

In the current paper, we demonstrate that BCG vaccination in genetically diverse DO mice provides significant protection upon *Mtb* challenge. This protection uniquely induces pathways associated with genes related to lung repair, particularly TGF-β receptor signaling and TLR10 pathway. The enhanced protection observed in BCG-vaccinated mice that also receive adjuvant correlated with improved formation of B-cell follicles, IL-17-producing CD4^+^ T-cell responses; lung transcriptional profiles accordingly show genes associated with B-cell responses, cellular responses to external stimuli as key pathways induced. Finally, we show that CD4^+^ T-cell responses mediate the enhanced protection in the lungs of DO mice vaccinated with BCG + adjuvant, as depletion of CD4^+^ T-cell responses reversed the enhanced protection as well as induction of immune activation and cytokine pathways. Together, our new data showcase that the DO mouse model of TB vaccination is a highly relevant model to probe early mechanisms of vaccine-induced protection and provide unreported, novel insights into lung pathways that mediate protection.

Consistent with other published studies using DO mice (6, 7), our results also show that BCG vaccination provides significant protection upon *Mtb* challenge. Although the protection is heterogeneous within the group of DO mice, BCG-vaccinated mice resulted in about 1 log reduction in *Mtb* CFU upon challenge, when compared with unvaccinated *Mtb*-infected mice. This protection is associated without a significant change in pulmonary inflammation or organization of lymphocytes within GrALT when compared to unvaccinated *Mtb*-infected lungs. Indeed, consistent with our published studies (2) where we examined lungs of DO mice at 50 dpi *Mtb* infection, even at 20 dpi, we observed significant enrichment of immune signaling genes as well as cytokine responses induced in lungs of *Mtb*-infected mice, suggesting that inflammatory and immune pathways, even in genetically diverse hosts, are induced within the first 20 dpi. Additionally, the bimodal distribution with respect to cytokine-producing CD4+ T cells in all groups infected with *Mtb*, and evident particularly with IL-22 (and to a lesser extent with IL-17), suggests the ability of individual DO mice to either become progressors or controllers. At the transcriptional level, there is significant overlap between *Mtb-*infected as well as BCG-vaccinated *Mtb*-infected lungs in that immune cell pathways and cytokine signaling are well represented in lungs of both groups of mice. Unique to the lungs of BCG-vaccinated mice following *Mtb* infection, we found that genes associated with lung repair, including TGF-β receptor signaling pathways, were induced in BCG-vaccinated *Mtb*-infected DO mouse lungs. Notably, Lars2 expression in B cells has been associated with TGF-β regulatory mechanism with mitochondrial aminoacyl-tRNA biosynthesis and programmed mitochondrial NAD^+^ regeneration and oxidative metabolism (20). Similarly, Retnla plays a role in lung repair and fibrosis (25), while Clca1 plays a role in mucus secretion and lung repair. Together, our data suggest that in DO mice that are BCG vaccinated, the induction of inflammatory genes that overlap significantly with *Mtb* infection is countered by induction of genes involved in lung repair. This is in stark contrast to this same group of genes that are downregulated in the context of *Mtb* infection alone, suggesting that BCG vaccination reverses the inhibition of lung repair genes that could contribute to enhanced *Mtb* control. Adjuvant treatment further enhanced the effect of BCG vaccination to promote lung repair via lung innate pathways early after *Mtb* infection.

Of the genes that are downregulated in the lungs of BCG-vaccinated *Mtb*-infected DO mice, we found that pathways involved in metabolism of proteins, TCA cycle and respiratory electron transport, and cell cycle were well represented. In addition, several genes involved in regulating transport of ions across the cell membrane and associated with regulation of TGF-β induced signatures were also downregulated in BCG-vaccinated *Mtb*-infected DO mouse lungs. Thus, although lung repair genes are induced in BCG-vaccinated lungs, there are still specific lung repair pathways that are further downregulated in the BCG-vaccinated lungs that regulate respiration and cell cycle.

We had previously shown that activation of lung innate pathways following BCG vaccination through the use of mucosal Amph CpG delivery (TLR9 agonist), along with CD40L activation and IL-10 neutralization, could significantly enhance protection upon *Mtb* challenge to afford complete control of *Mtb* (8). In the current study, we have replaced activation of lung innate mechanisms using MPL, a TLR4 ligand, rather than amph-CpG as an activator of innate immune responses. We show that activation of innate immune responses using MPL induced mDC accumulation in the lung, as well as activation of myeloid cells, which is reflected by upregulation of MHC Class II on monocytes, RMs, and mDCs. Consistent with our published studies, we also found that improved control coincided with increased accumulation of IL-17-producing CD4^+^ T cells. In contrast, while we found that IFN-γ-producing CD4 T-cell responses were also increased when amphCpG was used, we did not find IFN-γ-producing CD4^+^ T-cell responses to be increased when MPL was used for lung innate activation. Furthermore, both when amp-CpG was used in B6 mice and MPL was used in DO mice, we found enhanced accumulation of B-cell structures localized within GrALT as a feature that correlated with improved *Mtb* control in the BCG + adjuvant group. This coincided with pathways associated with translation, cellular responses to external stimuli, and mitochondrial translation, while the top genes induced in the lungs of this group were Jchain gene (immunoglobulin joining chain), MZB1 (marginal zone B and B1 cell-specific protein that promotes J-chain binding to IgA (31), and *S100A4* gene which is known to regulate Lck/Fyn activity in CD5 signaling to promote RORγt expression and Th17 polarization (32). These transcriptional profiles fit well with the induction of both Th17 cells as well as the formation of GrALT that is observed in the lungs of the BCG + adjuvant *Mtb*-infected DO mice. These data clearly suggest that during the early stage of immunity to *Mtb* infection, Th17 and GraLT responses, which have been described to be pathological in some other settings, can co-exist with, and likely promote lung repair and *Mtb* control. Interestingly, inflammation was reduced in BCG-adjuvant *Mtb*-infected mice that received CD4^+^ depletion antibody when compared with BCG + adjuvant *Mtb*-infected lungs, likely because CD4^+^ T cells can adversely contribute to inflammation. Thus, while we observe a correlation between the induction of GrALT and *Mtb* control in the BCG + adjuvant-treated group, we did not find that CD4+ T-cell depletion impacts GrALT formation (which only measures B-cell areas) in BCG + adjuvant *Mtb*-infected lungs. This is probably either because of the short period of depletion or the early time point post*-Mtb* infection studied here.

Finally, our results show that the improved protection, while resulting in activation of innate pathways, is mediated by the accumulation of CD4^+^ T cells. This is well reflected in the decreased induction of genes associated with the immune system, cytokine signaling in the immune system, IL-23 signaling, and stimulation by the CD28 family. Important cytokines that were reduced in this group were relevant cytokines such as *Il17a, Il17f*, *Ifng,* and *Il21*. Additionally, CD4 antigen expression was also reduced, confirming the depletion of CD4 T cells in the BCG-vaccinated + adjuvant group. Together, the transcriptional profile in the CD4-depleted BCG-vaccinated + adjuvant group demonstrated a clear lack of immune signaling, cytokine production, and reduction in chemokines, suggesting that while these are inflammatory mediators, they are likely driven by CD4^+^ T-cell response and mediate the improved protection observed in the BCG + adjuvant group. The use of adjuvants or innate immune modulators as described here is not translatable in the context of human vaccine settings, as it is not possible to predict the timing of exposure. However, the mechanistic insights gained by the animal studies, such as those described here, can help enhance our understanding of the correlates of protection that drive vaccine-induced immunity and delineate potential bottlenecks that need to be addressed for early and effective vaccine-induced immunity against TB.

The BCG-vaccinated DO mouse population showed a wide range of infection outcomes, similar to human disease, making them an improved small-animal model for studying TB infection and immunity. The DO mice also serve as sophisticated tools for mapping genes contributing to vaccination control, making them an important new resource in the fight against TB. In our previous study (2), we found that the expression of 16 ACS signature genes correlates with disease severity, even within the controller and progressor groups. The majority of human immune pathways induced in thenotlebly blood during TB disease progression are similarly induced in the lungs of DO mice and macaques, providing an in-depth understanding of conserved and diversified immune pathways in animal models and human TB. Notebly, across both the published study and here, we found that protection was associated with induction of genes associated with lung repair and TGF-β signaling. Additionally, our data for the first time show that while BCG vaccination can confer protective responses, these responses can be further improved upon by targeting the lung innate compartment, especially by induction of pathways associated with Th17 responses and B-cell responses. Whereas the administration of adjuvants targeting the lungs during infection is not directly translatable to human TB, these results enable us to start delineating a roadmap for the type of immune responses that a superior TB vaccine should induce, representing an important milestone in our mechanistic understanding of TB vaccine-mediated immune responses.

## MATERIALS AND METHODS

### Mice

DO mice (Jackson Laboratories, Bar Harbor, ME) were maintained under specific pathogen-free conditions at Washington University in St. Louis. Six-week-old female mice formed the various study groups. Studies were carried out with *n* = 25–56 individual mice per group for challenge studies (Tissues harvested day 20 post-*Mtb* challenge following vaccinations) (Fig. 1A).

### Vaccination and *Mtb* infection

The antigen protein Ag85B was prepared in-house as described in our previous work (38, 39). The adjuvant formulation comprised MPL-A (Avanti Polar Lipids, Alabaster, AL, USA) and FGK 4.5 (BioXcell, Lebanon, NH, USA). Anti-IL10r antibodies were purchased from BioXcell, Lebanon, NH, USA. For the depletion of CD4 T cells, αCD-40 antibodies were purchased from BioXcell, Lebanon, NH, USA. The bacterial stocks of BCG, *Mycobacterium bovis* BCG (BCG Pasteur, source: Trudeau Institute) and the strain *Mtb* HN878 (BEI Resources, Manassas, VA) were cultured to mid-log phase in Proskauer Beck medium containing 0.05% Tween80 and frozen as 1 mL aliquots at −80°C. Mice vaccinated with BCG received a single s.c. administration of 1 × 10^6^ CFU in 200 µL.

Following BCG vaccination, the adjuvant (Ag85B [10 µg + 50 µg MPL-A] + 100 µg FGK4.5]/mouse) was delivered intratracheally along with αIL-10r antibodies (800 µg/mouse), intraperitoneally, into the 6-week-old B6 mice 1 day prior to aerosol infection with *Mtb*, and a second dose of the formulation, 4 days post-infection. Four weeks after the BCG vaccination, mice were challenged by aerosol with a low dose (100 CFU) of *Mtb* HN878 (BEI Resources, Manassas, VA, USA) using the Inhalation Exposure System (Glas-Col, Terre Haute, IN, USA). Following infection, one group of mice was treated with anti-CD4 antibodies for CD4 depletion, while another group was treated with the isotype antibodies. BCG + adjuvant-treated mice received three doses of CD4 depletion on antibodies days −1 (1 day before infection) and weekly on days +4 and +11 following infection (300 μg/mouse, BioXcell clone GK1.5) until tissue harvest on 20 dpi. 20 days after the challenge, unvaccinated and vaccinated mice were sacrificed by CO_2_ asphyxiation, and the lungs were aseptically excised and individually homogenized in physiological saline solution. Serial dilutions of lung homogenates were plated on 7H11 agar for CFU and counted after 3 weeks of incubation at 37°C as described before (40).

### RNA-seq analysis

Lung tissue was homogenized, snap-frozen in RLT buffer, and DNase-treated total RNA was extracted using the Qiagen RNeasy Mini kit (Qiagen) as described before (2). cDNA libraries were prepared from RNA samples using PolyA selection, and processed cDNA was sequenced on the Illumina NovaSeq S4 platform (paired-end 150bp reads). Trimmomatic v0.36 (41) was used to trim adapters, and the STAR aligner (42) (v2.7.10a; 2-pass mode, basic) was used to align RNA-seq reads to the *Mus musculus* genome assembly (GRCm39, Ensembl release 107 (43). DESeq2 (v1.44.0) (11) was used to perform pairwise differential expression comparisons between data sets as described (uninfected, *n* =13; BCG, *n* = 8; BCG + adjuvant, *n* = 25; and BCG + adjuvant + CD4-depletion, *n* = 14) with the uninfected, unvaccinated sample set downloaded from a previous study (2) (*n* = 10). Genes were considered to be significantly differentially expressed when the FDR-corrected *P* value for upregulation or downregulation was ≤ 0.01. Principal components analysis was carried out using DESeq2 output (default settings). Differentially expressed gene sets across infected samples were intersected to identify gene sets of interest, and expression values across samples were visualized in heatmaps (MS Excel) using Z-scores of the relative expression level (FPKM) per gene. Pathway enrichment analysis for Reactome (12) pathways among gene sets of interest was performed using the WebGestalt (44) web server, using over-representation analysis to compare the enriched functions among gene sets, against a background of all the other Ensembl protein-coding genes, and affinity propagation to reduce redundancy in the results.

The raw RNA-seq read files (fastq) are accessible on the NCBI Sequence Read Archive (SRA [45], BioProject PRJNA523820). RNA-seq read counts and mapping statistics, as well as sample groupings and SRA accessions, are available for every sample in Table S1. Gene annotations, read counts, relative gene expression levels, and differential gene expression statistics for every gene and every sample comparison are provided in Table S2. Complete Reactome pathway enrichment results are provided in Table S3 and S5. Differential gene expression of the 13 mouse orthologs of the 16 ACS signature genes identified in Zak et al*.* (13) in the naive infected vs naive uninfected comparison is provided in Table S4.

### Generation of single-cell suspensions from tissues

Lung single-cell suspensions from *Mtb*-infected and vaccinated mice were isolated as previously described (46). Briefly, mice were euthanized with CO_2_, and lungs were perfused with heparin in saline. Lungs were minced and incubated in collagenase/DNase (Sigma-Aldrich) for 30 min at 37°C. Lung tissue was pushed through a 70 µm nylon screen to obtain a single-cell suspension. Red blood cells were lysed, and the cells were resuspended in suitable media or buffer for further use.

### Flow cytometry staining

For quantification of cytokine responses, cells were either stained immediately or stimulated with phorbol myristate acetate (PMA; 50 ng mL^−1^) and ionomycin (750 ng mL^−1^; Sigma Aldrich) in the presence of Golgistop (BD Pharmingen) as previously described (47). The following fluorochrome-conjugated antibodies were used for myeloid cell surface staining: CD11b APC (clone M1/70, Tonbo Biosciences), CD11c PE-Cy7 (clone HL3, BD Pharmingen), GR-1 PerCP-Cy5.5 (clone RB6-8C5, BD Pharmingen), and MHC class II FITC (clone M5/114.15.2, Tonbo Biosciences). CD3 AF700 (clone 500A2, BioLegend), CD4 Pacific Blue (clone RM4.5, BD Pharmingen), and CD44 PE-Cy7 (clone IM7, Invitrogen) were used for T-cell surface staining. For intracellular staining, fixation/permeabilization concentrate and diluent (BD Biosciences) were used to fix and permeabilize lung cells following the manufacturer’s instructions. Intracellular staining with IFN-γ APC (clone XMG1.2, Tonbo Biosciences), IL-17A FITC (clone TC11-18H10, BD Pharmingen), IL-22 PE (clone 1H8PWSR, BioLegend), or the respective isotype control antibodies (APC rat IgG1κ, FITC rat IgG1κ, and PE rat IgG1κ isotype, BD Pharmingen) was performed for 30 min at 4 °C. Cells single-stained with each fluorochrome were used as controls for the compensation matrix in the flow cytometry. Cells were processed with the Becton Dickinson (BD) Fortessa X-20 flow cytometer using FACS Diva software, or the BD FACSJazz flow cytometer using FACS software (BD). Flow cytometry experiments were analyzed using FlowJo (Tree Star Inc.). The representative gating strategies (46–49) are represented in Fig. S2.

### Histological analysis

The upper right lung lobe was perfused with 10% buffered formalin and excised from mice for histological analysis of inflammation. The excised lobes were embedded in paraffin, and sections (5 µm) of FFPE lung were cut using a microtome, stained with H&E, and processed for microscopy. Images were captured using the automated NanoZoomer digital whole-slide imaging system (Hamamatsu Photonics). Regions of inflammatory cell infiltration were delineated utilizing the NDP view2 software (Hamamatsu Photonics), and the percentage of inflammation was calculated by dividing the inflammatory area by the total area of individual lung lobes. All scoring was conducted in a blinded manner utilizing *n* = 25–56 mice per group.

### Blinded morphometric analysis of B-cell follicles as a measure of GRALT

Lung sections were stained with CD45R/B220 (47) to identify distinctive B-cell follicles, which are an essential component of GRALT. All B-cell follicles were blindly outlined with an automated tool of the Zeiss microscope to calculate the area of GRALT in experimental groups.

### Statistical analysis

Statistical analyses of all data were performed using GraphPad Prism (La Jolla, CA, USA). One-way ANOVA with Tukey’s correction test or unpaired two-tailed Student’s *t*-test was done for comparisons among the groups. For correlation analysis, Pearson’s correlation coefficient was used. Multiple linear regression analysis for correlations of immune cells with bacterial burden was performed in R (v4.4.0) using the stats package for model fitting and dplyr/readr for data handling. Regression coefficient significance was tested using a two-sided t-test compared to β = 0. Pairwise correlations between predictors were calculated using Pearson’s correlation coefficient. Collinearity was assessed with VIFs using car (where values <5 are considered acceptable). Nested models were compared using likelihood ratio tests (run in the base R ANOVA function), which assess whether adding predictors significantly improves model fit. The test compares the difference in log-likelihoods of the two models to a χ² distribution with degrees of freedom equal to the difference in model parameters.

## Data Availability

The raw RNA-seq read files (fastq) are accessible on the NCBI Sequence Read Archive (SRA [45] BioProject PRJNA523820). RNA-seq read counts and mapping statistics, as well as sample groupings and SRA accessions, are available for every sample in Table S1. Gene annotations, read counts, relative gene expression levels, and differential gene expression statistics for every gene and every sample comparison are provided in Table S2.

## SUPPLEMENTAL MATERIAL

Figure S1 mbio.03232-25-s0001.tif APC flow cytometry.

Figure S2 mbio.03232-25-s0002.tif Hierarchical gating strategy.

Tables S1 to S5 mbio.03232-25-s0003.xlsb Bioinformatic data.

Graphical Abstract mbio.03232-25-s0004.tiff Summary.

ASM does not own the copyrights to Supplemental Material that may be linked to, or accessed through, an article. The authors have granted ASM a non-exclusive, world-wide license to publish the Supplemental Material files. Please contact the corresponding author directly for reuse.
