# Supplementary figures and images for: Targeting the lung innate pathways during tuberculosis can improve vaccine-induced protection via Th17 responses in diversity outbred mice
Source: mBio. 2026 Jan 20;17(2):e03232-25. doi: 10.1128/mbio.03232-25 (PMC12893010; doi:10.1128/mbio.03232-25)

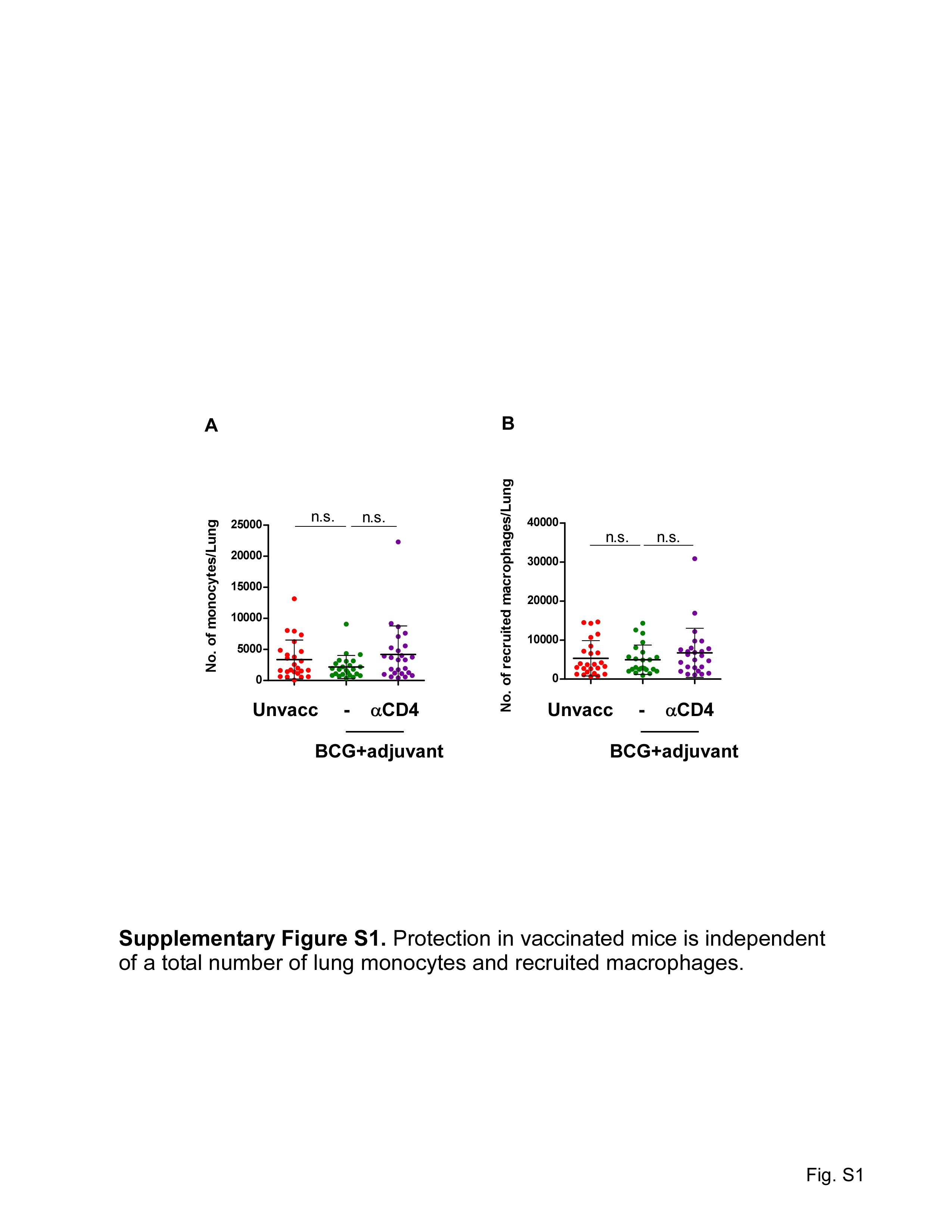

Supplement: Figure S1 — APC flow cytometry. [file mbio.03232-25-s0001.tif]

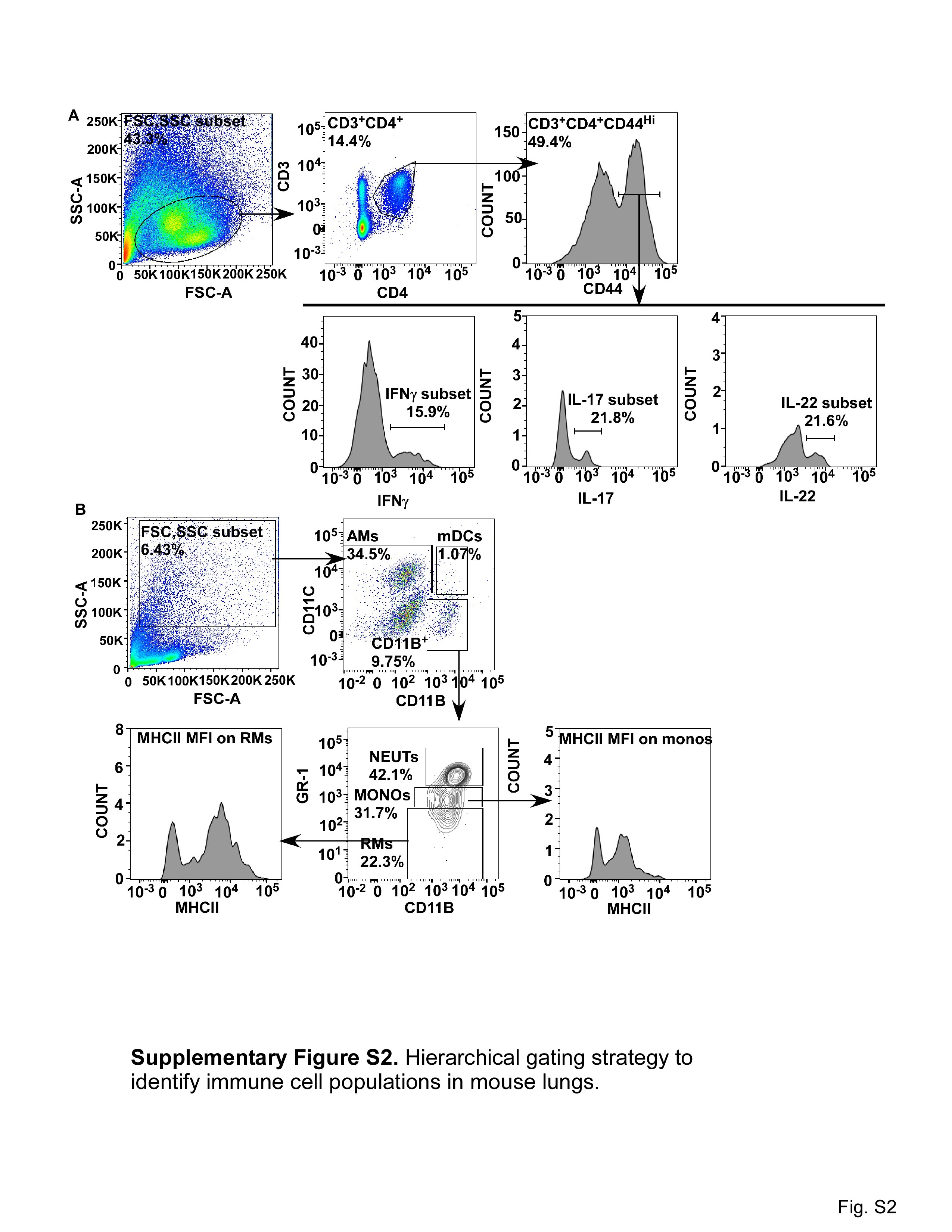

Supplement: Figure S2 — Hierarchical gating strategy. [file mbio.03232-25-s0002.tif]

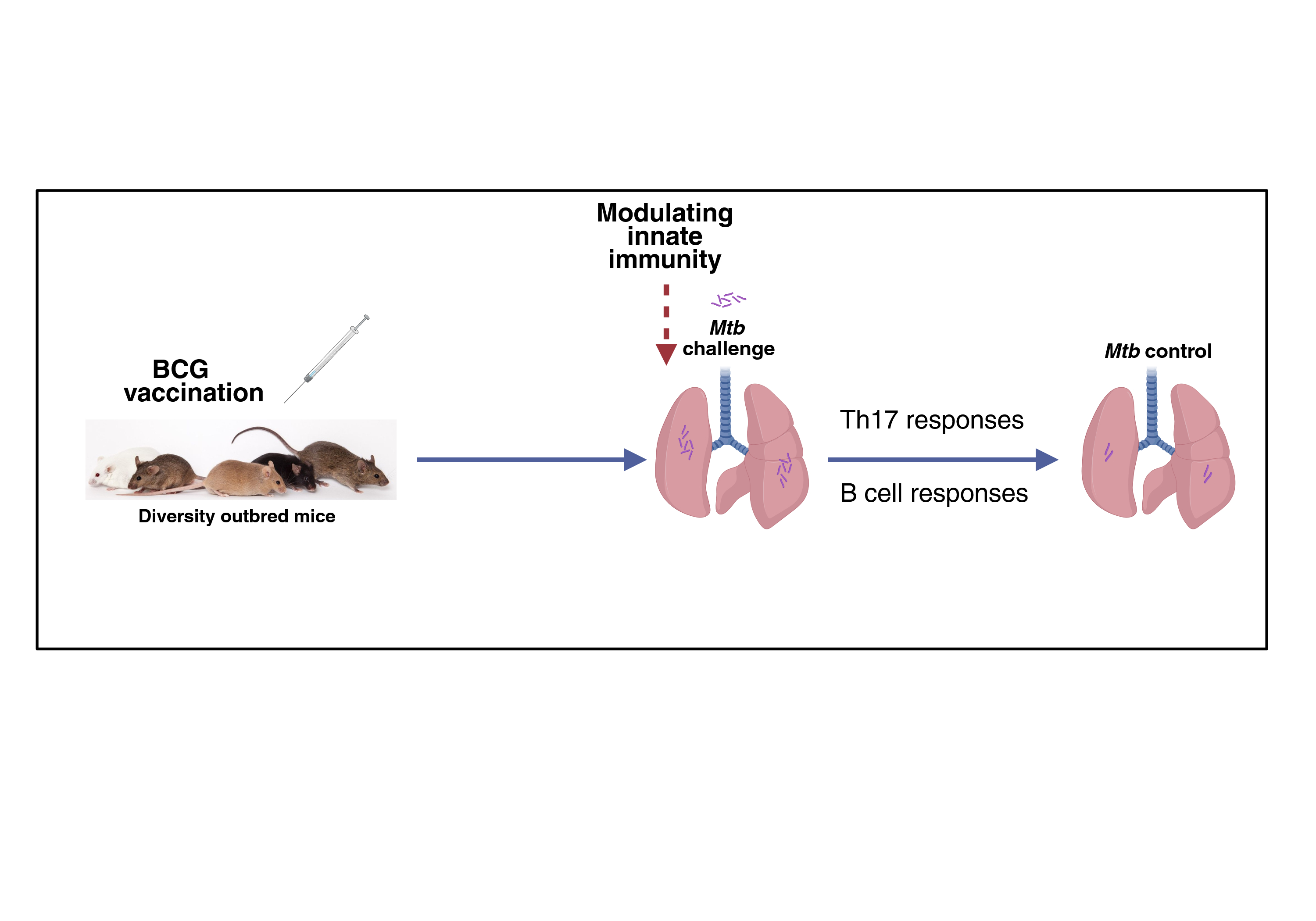

Supplement: Graphical Abstract — Summary. [file mbio.03232-25-s0004.tiff]
